# Signal-to-noise ratio evaluation of magnetic resonance images in the presence of an ultrasonic motor

**DOI:** 10.1186/s12938-017-0331-1

**Published:** 2017-04-14

**Authors:** Peyman Shokrollahi, James M. Drake, Andrew A. Goldenberg

**Affiliations:** 1 Institute of Biomaterials and Biomedical Engineering, University of Toronto, Rosebrugh Building, 164 College Street, Room 407, Toronto, ON M5S3G9 Canada; 2 Division of Neurosurgery, The Hospital for Sick Children, 555 University Avenue, Room 1504, Toronto, ON M5G1X8 Canada; 3 Department of Mechanical and Industrial Engineering, University of Toronto, 5 King’s College Road, Room 106, Toronto, ON M5S3G8 Canada; 4 123 Homewood Avenue, Toronto, ON M2M1K2 Canada

**Keywords:** Magnetic resonance imaging, Ultrasonic motor, Signal-to-noise ratio, Deflection torque, MRI-compatibility

## Abstract

**Background:**

Safe robot-assisted intervention using magnetic resonance imaging (MRI) guidance requires the precise control of assistive devices, and most currently available tools are rarely MRI-compatible. To obtain high precision, it is necessary to characterize and develop existing MRI-safe actuators for use in a high magnetic field (≥3 T). Although an ultrasonic motor (USM) is considered to be an MRI-safe actuator, and can be used in the vicinity of a high field scanner, its presence interferes with MR images. Although an MR image provides valuable information regarding the pathology of a patient’s body, noise, generally of a granular type, decreases the quality of the image and jeopardizes the true evaluation of any existing pathological issues. An eddy current induced in the conductor material of the motor structure can be a source of noise when the motor is close to the isocenter of the image. We aimed to assess the effects of a USM on the signal-to-noise ratio (SNR) of MR images in a 3-T scanner. The SNR was compared for four image sequences in transverse directions for three orientations of the motor (x, y, and z) when the motor was in the “off” state. The SNR was evaluated to assess three artifact reduction methods used to minimize the motor-induced artifacts.

**Results:**

The SNR had a range of 5–10 dB for slices close to the motor in the x and y orientations, and increased to 15–20 dB for slices far from the motor. Averaging the SNR for slices in all cases gave an SNR loss of about 10 dB. The maximum SNR was measured in the z orientation. In this case, the SNR loss was almost the same as that of other motor orientations, approximately 10 dB, but with a higher range, approximately 20–40 dB.

**Conclusions:**

The selection of certain scanning parameters is necessary for reducing motor-generated artifacts. These parameters include slice selection and bandwidth. In developing any MRI-compatible assisted device actuated by a USM, this study recommends the use of an approximately 3-mm slice thickness with minimum bandwidth to achieve optimized SNR values when a USM is operating close to (within approximately 40 mm) the region being imaged. The SNR can be further enhanced by increasing the number of signal averages, but this is achieved only at the cost of increased scan duration.

## Background

Magnetic resonance imaging (MRI)-compatible robots are expected to safely and efficiently revolutionize surgical operations by enhancing dexterity in a digitized imaging environment and providing precise positioning tools in real time [1]. These robots can overcome human limitations of dexterity and stamina by delivering superior spatial resolution and geometric accuracy [2], while also exploiting the versatile modalities and functionalities of MRIs. To perform a safe surgical procedure with high precision and full controllability, the robot must have precise actuators that operate within the magnetic fields of the MR environment as accurately as they can outside it without affecting image quality [3, 4].

However, because of the limitations imposed by MRI magnetic fields, numerous challenges exist when using various medical tools and devices. Safety standards limit the accessibility of many devices and MRIs for many patients because of the possibility of harm. As reported in Scientific Reports in 2015, approximately 300,000 patients are denied MRIs each year because of these limitations [5].

Research on MRI-compatible robotic systems illustrates that recently developed MRI-compatible actuators are based on ultrasonic, pneumatic, and hydraulic systems. Other types, such as AC or DC actuators, which are commonly used in robotics, have been tested and rejected for MRI because of their high interaction with the magnet due to the ferromagnetic materials that they contain [1]. Pneumatic and hydraulic actuators are decoupled from electro-magnetism by using MRI-compatible materials. However, hydraulic actuators are rarely used because of their high risk of leakage in cases in which sterility is critical, and also because of their inherent controllability issues [6]. Pneumatic motors also suffer from issues of stability, controllability in motion, and high complexity, while requiring tightly geared break mechanisms to lock the system in a malfunction case [7, 8]. Ultrasonic motors (USMs) have the advantages of high accuracy, high torque/size ratio, compactness, and non-back-drivability (i.e., they cannot move when turned off). Although these motors suffer from interference with MRI magnets and noise generation during motion and while motionless, they are considered to be the most promising actuators because of their high controllability and accuracy. Noises are generated by the power and control signals that are transferred to the motor and by an eddy current induced in the conductor materials within the motor structure, and attachments such as cables or an encoder.

The focus of this research is to determine the effects of a USM on MR images in the high field MRI scanner (3T) using signal-to-noise ratio (SNR). The SNR is a metric used to quantify MR image quality [9]. The signal is defined as the mean pixel intensity within the measurement region of interest (MROI) in the phantom image. The noise is defined as the random variation in pixel intensity induced by the USM within MROI, and deteriorates the MR image quality [10, 11]. Because SNR evaluation of the motor in the “on” state has already been performed [12], here we consider the SNR degradation of the image when the motor is off.

The SNR is affected by such factors as the volume of the voxel, the bandwidth, and the number of signal averages (NSA). The following formula illustrates the relationship between the SNR and these factors [13].

1 $$SNR = K (Voxel Volume) \sqrt {\frac{NSA}{Bandwidth}}$$

To compensate the SNR for cases in which the USM is close to the isocenter, bandwidth decrements and slice thickness increments are evaluated in the present research.

**Fig. 1:**
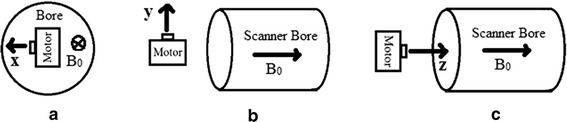
Motor orientations with respect to the scanner bore, **a** x, **b** y, and **c** z

This study investigates the SNR values of images in the presence of a USM for three orientations of the motor inside the magnet bore (Fig. 1) and for the “off” states, presented in the “Methods” section. The results are presented in the “Results” section. The significance of the results and findings are discussed in the “Discussion” section, and a brief conclusion, which integrates the results and discussion, is presented in the “Conclusion” section.

## Methods

A USM, PUMR 40E (Piezoelectric Technology Co., Ltd., Seoul, Republic of Korea), was used to evaluate SNR in a 3T Achieva scanner (Philips Healthcare, Best, NL). The best image orientation to cover the noise distribution was transverse (along the plane perpendicular to the foot-head axis), as noise was distributed uniformly through the center of image. The SNRs for the coronal and sagittal planes were not evaluated because of their nonuniform noise distribution. The motor and a phantom were placed in a 32-channel head coil (Philips) inside the scanner. The motor was placed close to the center of the phantom. Thus, the most noise was observed in the middle of the circular cross-section of the phantom.

The single-image method and double-image method SNR evaluations were used [11]. In the single-image method, the signal mean was calculated by averaging the MROI pixels’ intensities, a set of pixels centered in a square box with the maximum size fitted into the area of the phantom image (whichever is larger). The standard deviation (SD) of the noise was calculated from pixels located in the background of the image, i.e., the non-signal producing region at the corner and outside the phantom image. The SNR was calculated by dividing the signal mean by the standard deviation of the noise.

In the double-image method, images of the uniformity phantom were acquired twice. First, the scan was performed when the USM was absent. The images of this scan are referred to as reference images or Image1. Another scan was performed with the USM present. The images of this sequence are referred to as signal images or Image2. By subtracting the reference image from the signal image, noisy images or Image3 were generated. The mean value of the MROI pixels’ intensity was measured in the reference images. The SD of the noise was calculated from the noisy image (Image3) after subtracting two identically scanned images:

2 $${\text{Image3}} = {\text{Image2}} - {\text{Image1}}$$

where Image2 is the image of the phantom when the motor is present and Image1 is the image of the phantom when the motor is absent. The SD was calculated within the MROI pixels of Image3, and was corrected by dividing by $\sqrt 2$.

### SNR evaluation for common clinical image sequences

**Fig. 2:**
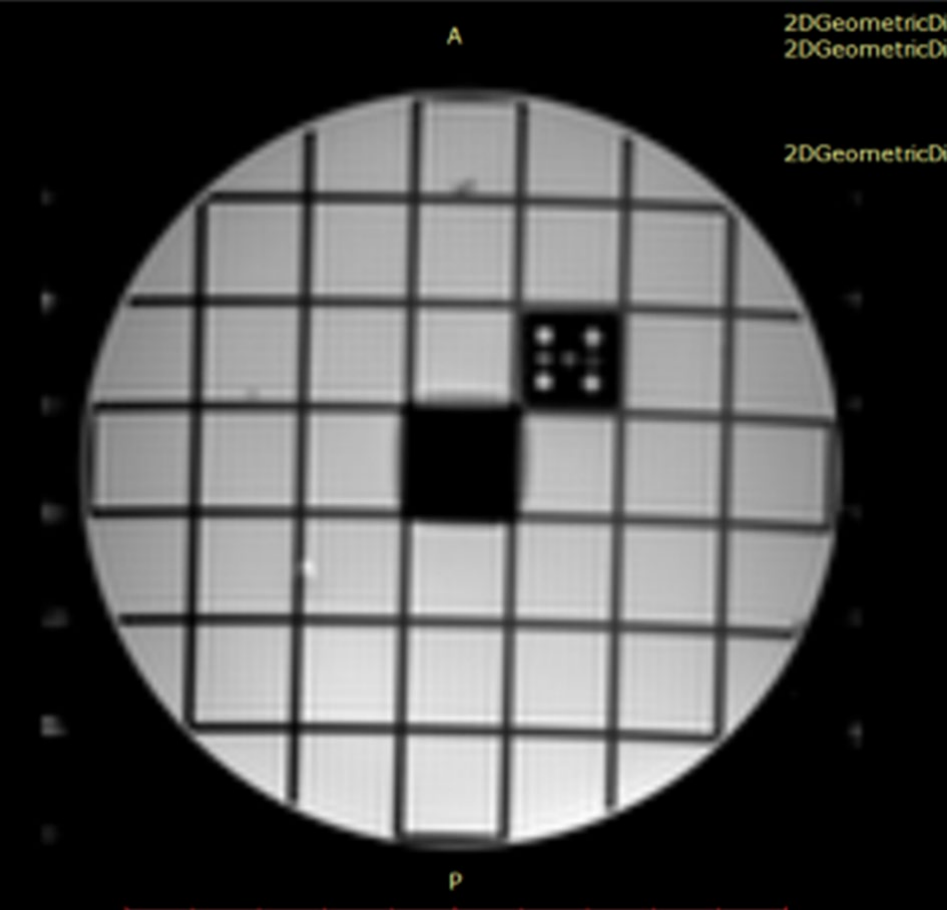
Appearance of “ghosts” in the background

In addition, imaging filters were avoided, because these filters might have different effects at the center and edges of images where the signal and noise are measured respectively [14]. Figure 2 illustrates the appearance of “ghosts” in the background (dots on the left and right side of the phantom image) for the T1-weighted sequence, when the motor was not present. As ghost noise was observed in the background, the double-image method was selected to evaluate the SNR.

MATLAB 2013b was used to evaluate the SNR and plot the noisy image for the abovementioned double-image procedure. The maximum MROI was selected for each scan to include the maximum amount of the noise generated by the motor. The SNR was evaluated for three motor orientations: x, y, and z.

The following sequences were applied: T1-weighted spin echo (T1W), T2-weighted turbo spin echo (T2W), fast spin echo (TSE), and gradient echo (FFE). The images were acquired according to the application of common clinical imaging specifications as follows:

- T1W: TE = 10 ms, TR = 0.60 s, FOV = 160 × 160 × 150 mm, in-plane voxel size = 1 mm, slice thickness = 5 mm, and flip angle = 70°.
- T2W: TE = 80 ms, TR = 3 s, FOV = 160 × 160 × 150 mm, in-plane voxel size = 1 mm, slice thickness = 5 mm, flip angle = 90°, and turbo factor = 5.
- TSE: TE = 72 ms, TR = 4 s, FOV = 160 × 160 × 150 mm, in-plane voxel size = 1 mm, slice thickness = 5 mm, flip angle = 90°, and turbo factor = 16.
- FFE: TE = 2.8 ms, TR = 12.1 ms, FOV = 160 × 160 × 150 mm, in-plane voxel size = 1 mm, slice thickness = 5 mm, and flip angle = 30°.

### SNR evaluation for artifact compensation techniques

Because a reduction in slice thickness and a bandwidth increment are common compensation methods for a reduction of artifacts, the SNR was evaluated to compare the performance of these factors on improving the image quality. To evaluate the impact of both slice thickness and bandwidth increments on the SNR, three slice thicknesses (1, 3, and 5 mm) and two bandwidths (437 and 875 Hz) were tested. Because the voxel size is directly proportional to the slice-thickness, Eq. (1) can be used to anticipate the effects of the slice thickness on the SNR.

Equation (1) indicates that the SNR is increased by a factor of $\sqrt 2$ when the bandwidth is halved. In addition, by decreasing the slice thickness from the initial value of 5 mm to 3 mm, and then to 1 mm, the SNR is reduced to 60 and 20% of its initial value, respectively.

Because avoiding parallel image techniques resulted in the removal of aliasing in the periphery of the image background, the single-image method was used for SNR evaluation using the RadiAnt DICOM Viewer 2.2.9 software package. The four abovementioned sequences were employed with the following image specifications:

- T1W: TE = 10 ms, TR = 0.60 s, FOV = 160 × 160 × 150 mm, in-plane voxel size = 1 mm, slice thickness = 5 mm, and flip angle = 70°.
- T2W: TE = 80 ms, TR = 3 s, FOV = 160 × 160 × 150 mm, in-plane voxel size = 1 mm, slice thickness = 5 mm, flip angle = 90°, and turbo factor = 1.
- TSE: TE = 72 ms, TR = 4 s, FOV = 160 × 160 × 150 mm, in-plane voxel size = 1 mm, slice thickness = 5 mm, flip angle = 90°, and turbo factor = 1.
- FFE: TE = 2.8 ms, TR = 12.1 ms, FOV = 160 × 160 × 150 mm, in-plane voxel size = 1 mm, slice thickness = 5 mm, and flip angle = 30°.

To evaluate the impact of both slice-thickness reduction and bandwidth increment on SNR, three slice thicknesses (1, 3, and 5 mm) and two bandwidths (437 and 875 Hz) were tested. The obtained SNR values were compared, as were also the distortion ratio and image artifact size.

## Results

### SNR evaluation for common clinical image sequences

**Fig. 3:**
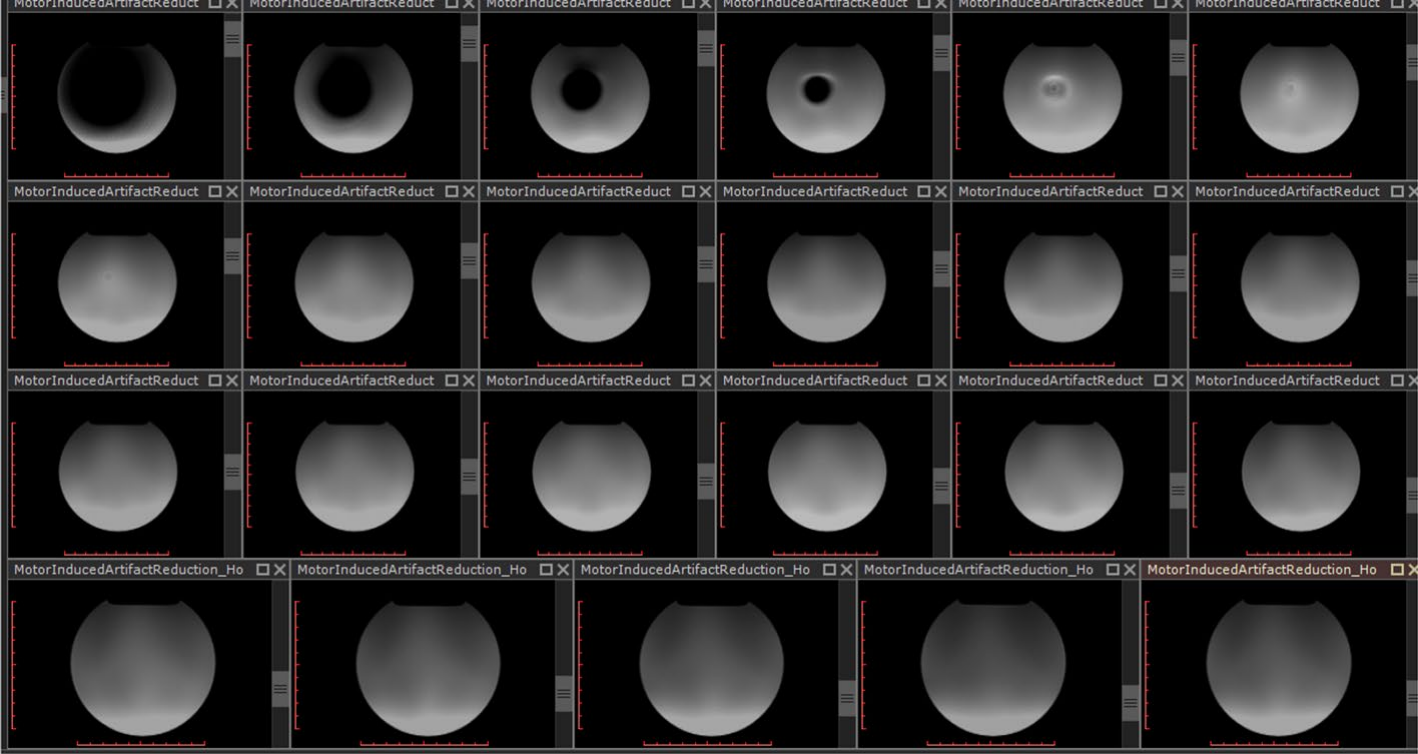
A complete T1W image sequence illustrating the slice location of signal voids and pileups

**Fig. 4:**
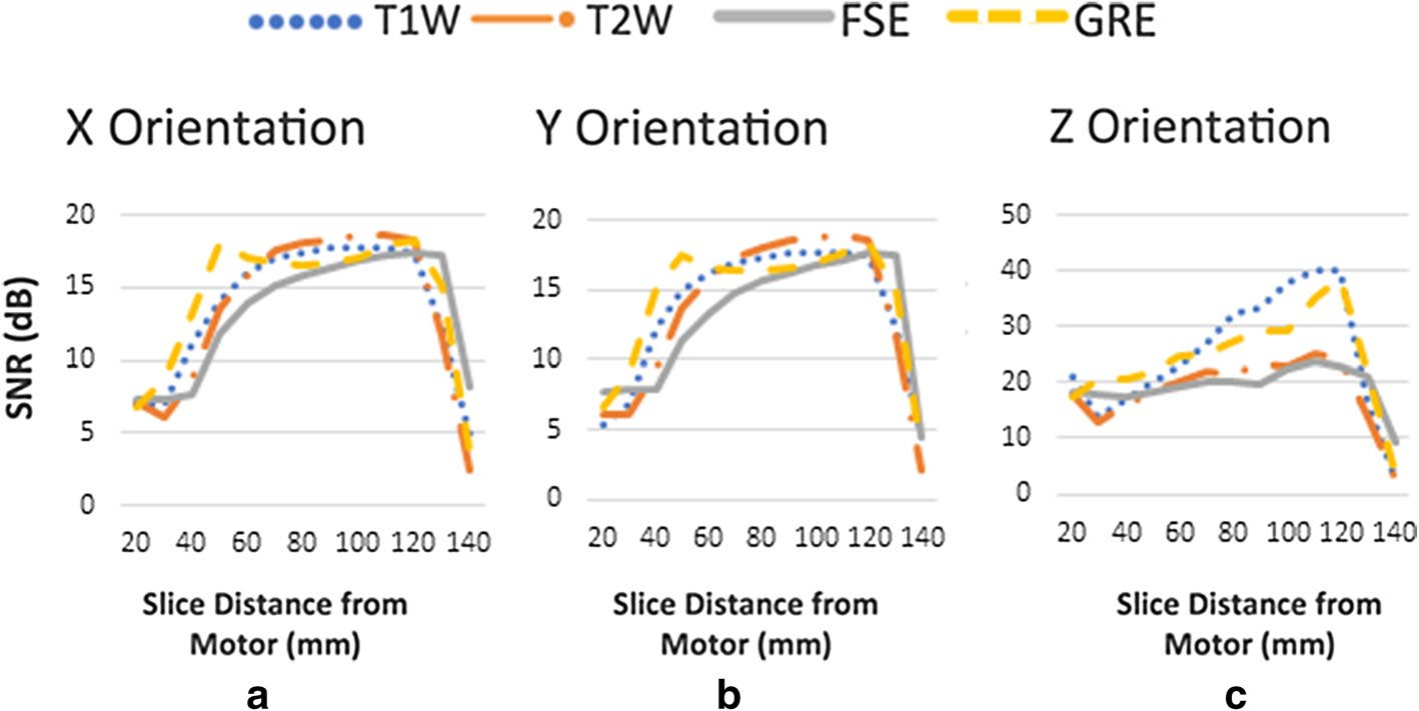
SNRs when the motor was in the **a** x, **b** y, and **c** z orientations

Figure 3 illustrates a sequence of T1W signal images. In the first slices, signal voids result in low values for signal intensity means. Signal voids were present in images up to 40, 40, and 30 mm away from the motor’s center of mass for the x, y, and z orientations respectively. Pileups are present at 50, 50, and 40 mm away from the motor for the x, y, and z orientations respectively. The SNR values were calculated when the motor was in three orientations (Fig. 4).

### SNR evaluation for artifact compensation techniques

#### Comparison of SNR values in various image sequences

**Fig. 5:**
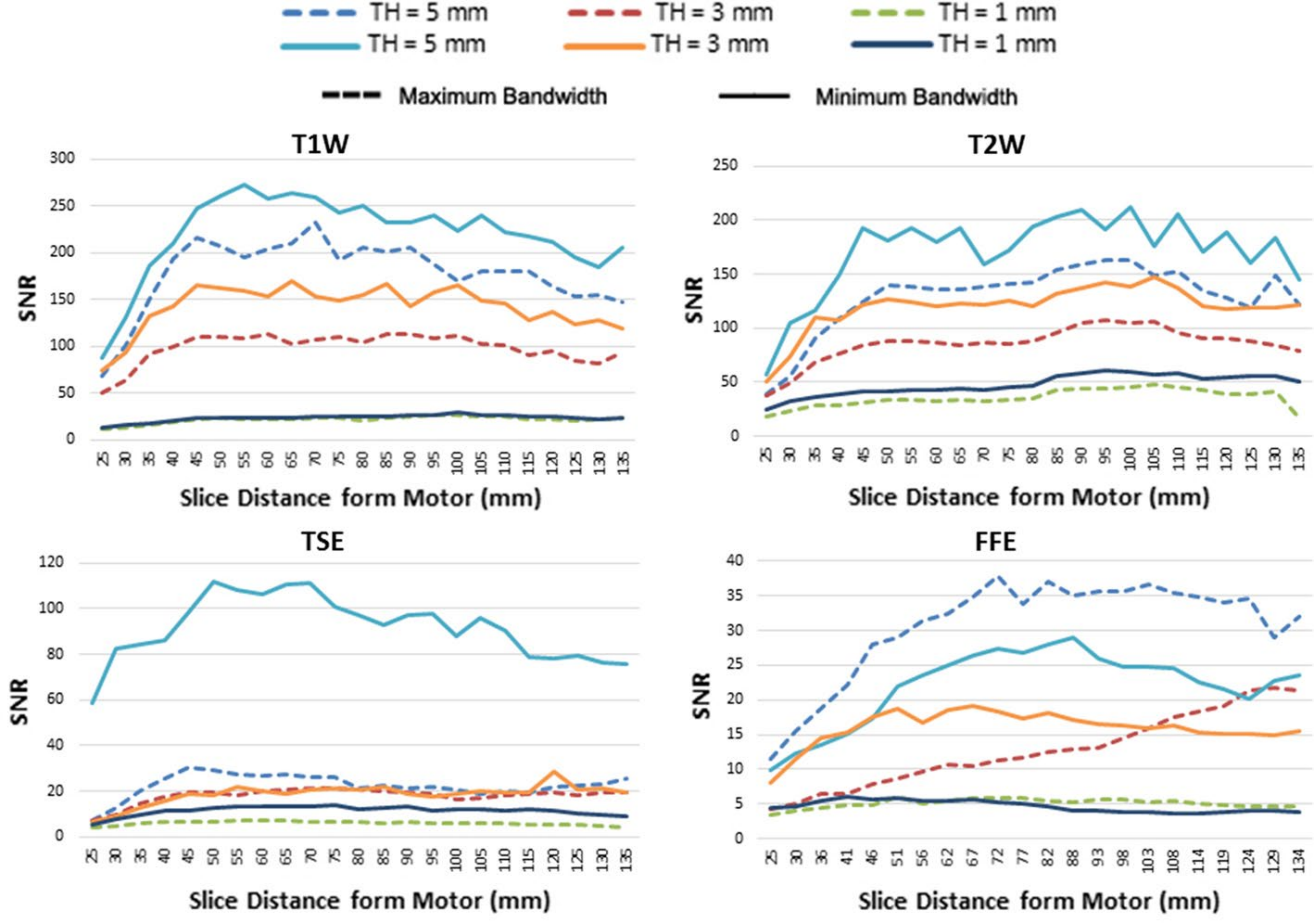
Comparison of SNR values for three slice thicknesses

The SNR values were compared for three slice thicknesses (TH) and the minimum and maximum bandwidth for the four outlined sequences. Figure 5 illustrates the comparison of SNR values for the three slice thicknesses, and the minimum bandwidth (solid lines) and maximum bandwidth (dashed lines) for T1W, T2W, TSE, and FFE sequences. Figure 5 shows that the FFE has low SNR values compared to the other three image sequences, and that T1W has the maximum SNR.

**Fig. 6:**
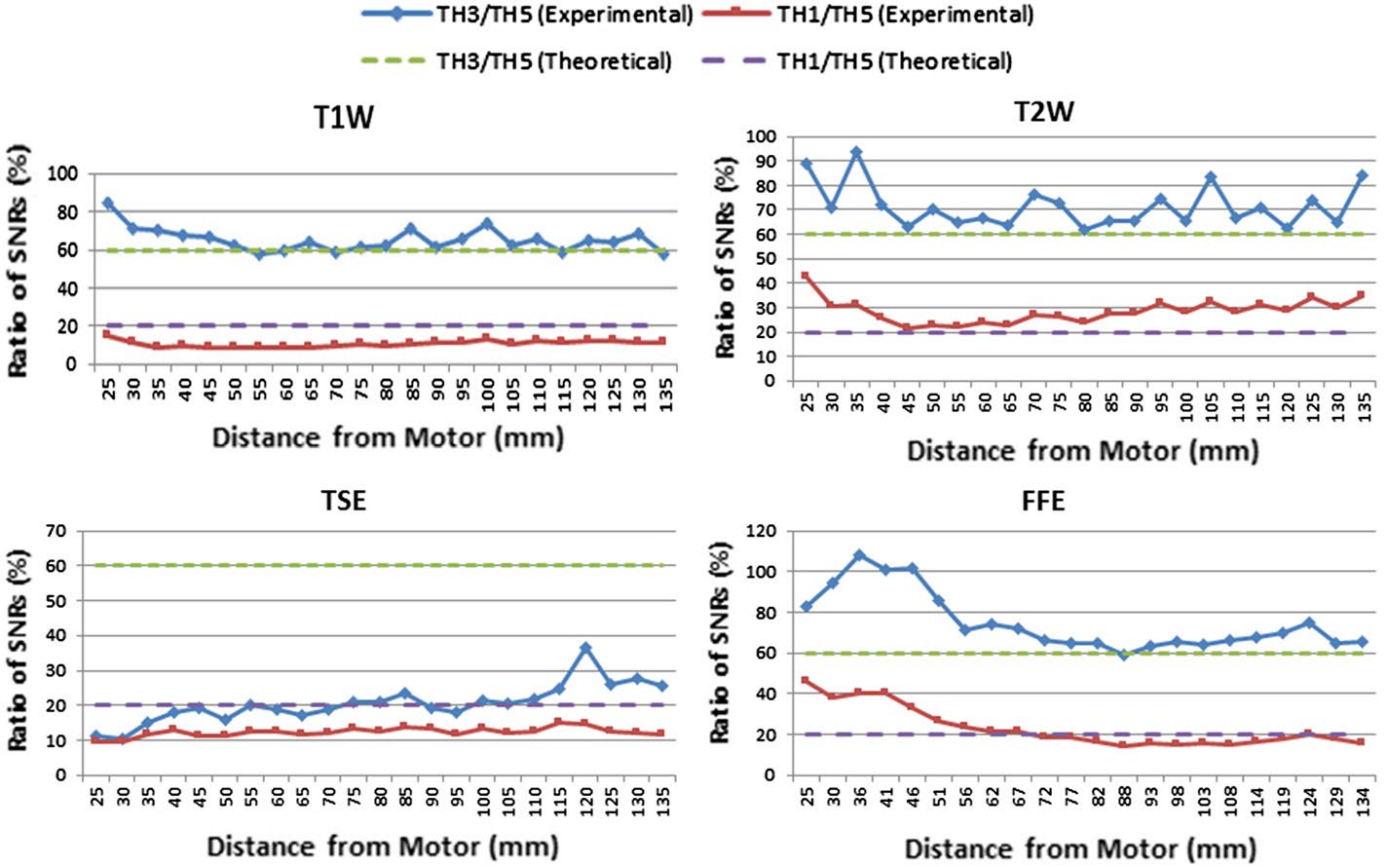
Ratio of SNR values for different slice thicknesses

Halving the bandwidth increased the SNR by approximately 30 to 40%. However, it had an insignificant effect on the SNR for TH = 1. By decreasing the slice thickness from the initial value of 5 to 3 mm, and then to 1 mm, the SNR was reduced to 60 and 20% of its initial value, respectively. Figure 6 illustrates the ratios of the SNR for slice thicknesses of 3–5 mm, and 1–5 mm, along with the anticipated theoretical values. In this figure, the experimental values are in solid lines and the theoretical ones are in dashed lines for T1W, T2W, TSE, and FFE.

#### Comparison of SNR values along with motor-induced geometric distortions

Table 1 shows the range of geometric distortion ratio along with the corresponding SNR for the T1W sequence, when the motor was in the y orientation. When comparing the maximum distortion ratio for the minimum and maximum bandwidth as shown in Table 1, the geometric distortion ratio almost halves when the bandwidth is doubled. Although decreasing the slice thickness does not affect the geometric distortion ratio, it decreases the SNR values. The results in Table 1 are consistent for all four sequences (T1W, T2W, TSE, and FFE). The ranges of distortion ratio are the same for all sequences but the SNR degrades, with the highest value for T1W then lowering in the order T2W, TSE, and FFE.

**Table 1:** Range of geometric distortion ratio and SNR for T1W sequence

TH (mm)	BW		Distance from motor (mm)	
	Minimum	Maximum	Minimum	Maximum
5	4–0.7% (SNR: 186–259)	2.3–0.9% (SNR: 150–232)	37	67
3	5.5–1.7% (SNR: 132–154)	2.9–1.1% (SNR: 92–112)	34	58
1	3.7–1.7% (SNR: 13–23)	2.9–1.5% (SNR: 11–21)	28	44

#### Comparison of SNR values along with motor-induced artifact size

The range of artifact size was compared for the four abovementioned sequences for three slice thicknesses and two bandwidths. Table 2 shows the results for the T1W sequence. The slice thickness reduction reduces the size of artifacts, but the bandwidth increment does not significantly change the size of artifacts. A similar SNR relationship was obtained for T1W and the other sequences discussed above. Table 3 shows a summary of all comparisons of artifact reduction methods.

**Table 2:** Range of artifact size for T1W sequence, y orientation

TH (mm)	BW		Distance from motor (mm)	
	Minimum	Maximum	Minimum	Maximum
5	12.5–46.4% (SNR: 87–248)	7.4–46.3% (SNR: 68–216)	25	45
3	2.3–26.8% (SNR: 74–143)	2.1–26.4% (SNR: 50–100)	26	38
1	4.9–18.4% (SNR: 13–15)	3.4–17.0% (SNR: 11–13)	26	29

**Table 3:** Compensation method impacts

Sequence	TH (mm)	Artifact size impact				BW impact on geometric distortion						Acquisition duration (min)
		SNR and distance	Artifact size (%)		Distance range (mm) for present artifact	SNR and distance	Min/max BW				Distance range for geometric distortion	
							Min BW		Max BW			
			Max	Min			Max dist (%)	Min dist (%)	Max dist (%)	Min dist (%)		
T1W	3		26.4%	2.1%			5.5%	1.7%	2.9%	1.1%		3:15
		SNR	50	100		SNR	132	154	92	112		
		Distance from motor (mm)	26	38	12	Distance from motor (mm)	34	58	34	58	24	
T2W	3		31.6%	4.8%			5.2%	1.8%	2.3%	0.9%		1:39
		SNR	37	77		SNR	110	122	68	85		
		Distance from motor (mm)	26	40	14	Distance from motor (mm)	34	46	34	46	12	
TSE	3		31.4%	6.7%			4.3%	1.7%	2%	1.4%		3:12
		SNR	7	14		SNR	9	18	10	20		
		Distance from motor (mm)	26	35	9	Distance from motor (mm)	31	49	31	49	18	
FFE	3		39.3%	13.5%			3.1%	2%	1.5%	1.2%		1:33
		SNR	4	6		SNR	8	19	4	9		
		Distance from motor (mm)	27	40	13	Distance from motor (mm)	26	48	26	48	22	

## Discussion

As Eq. (1) shows, the SNR depends on three factors: the bandwidth, voxel volume, and NSA. However, the SNR does not depend on the other factors related to the sequence, such as T1 and T2. The differences among the results obtained for T1W, T2W, and TSE, which are considered to be the same type, are due to the difference between the quality factor related to the type of scanner used, and the type of RF coil applied. A comparison shows that T1W has a higher SNR than T2W for most of the different MRI scanner vendors investigated [15].

As shown in Fig. 6, for T1W, T2W, and FFE, the ratio is higher than expected in slices where motor artifacts are present. Larger SNR values than anticipated were obtained. This indicates that a reduction in the slice thickness to reduce image artifacts generated by a motor has a lesser effect on decreasing the SNR values. Because this ratio to a certain extent reaches a value of about 90%, we recommend the use of an approximately 3-mm slice thickness when a motor is present within the vicinity of the isocenter.

The differences between experiments and simulation in the TSE and FFE sequences are bigger than in the T1W and T2W sequences (Fig. 6). This is due to the difference between image sequences. The SNR values obtained for TSE and FFE are lower than those for T1W and T2W. Some of the known parameters that may cause this difference are echo time (TE), repetition time (TR), and flip angle. These parameters are significantly different, especially for FFE.

Other factors affecting SNR values include static magnetic field strength, radiofrequency coil, proton density (PD), slice gap, matrix size, field of view, NSA, and parallel imaging. In SNR evaluation for artifact compensation techniques, all these factors were the same for all sequences. Parallel imaging was also avoided.

The use of the double-image method increases the total scanning time by a factor of 2. This method may be susceptible to the MRI’s system drift, in which the quality of the image may change when the scanner is used for a long time. However, because the time between scans was on the order of a few minutes, the system drift was nonsignificant. It has been reported that in the absence of system drifts, this method can be considered the most reliable SNR evaluation technique [14].

Although the single-image method is faster, more clinically common, and more robust to system drift than the double-image method, we used the double-image method because the single-image method is susceptible to subtle artifacts that can interfere with the noise measurements and cause anomalous results. Therefore, because we observed ghost noise in the background, the double-image method was employed for SNR evaluation of images scanned with common clinical specifications. Because double imaging method was avoided when comparing reduction methods, the single-imaging method was used in this case and resulted in significantly reducing the scanning time.

The source of the noise is an eddy current that the MR gradient fields induce in the conductive parts of the motor. Inversely, the motor is able to induce eddy currents in MR metallic components such as the receiver coil. In addition, the motor further induces eddy currents in the MR coil when it is on and the shaft is rotating. The transmission lines of the USM can also induce RF noise. The effect on image degradation when the motor is on has been studied by Chinzei [12]. The current induced by the motor in the scanner body and the receiver coil can be another source of noise.

The important factors affecting the SNR of the images acquired with common clinical specifications were the parallel imaging acquisition, turbo factor, and slice thickness. In these images, the low SNR observed from the first slices, which had signal voids and pileups (Fig. 3), is because of low mean signal values. After these slices, the SNR reaches a steady state value. The SNR decrease in the last few slices is because of the presence of air in the voxels close to the end of the phantom.

The values of the SNR for the z orientation are higher than the other two orientations as the motor is symmetrical to the static magnetic field, thus it perturbs the MRI fields to a lesser extent. This is consistent with other results in the image artifact analysis.

Signal and noise measurements depend significantly on scan parameters and test conditions. Parameters that have been reported to affect the SNR include pulse sequence, BW, slice thickness, and receiver coil [16]. This research, concerned with the effects of the presence of a motor near the image region of interest, shows that slice thickness reduction and bandwidth increment decrease the SNR (Table 3).

Scanning duration is a critical factor for consideration in MRI. There is a trade-off between speed of acquisition and image quality [14]. These factors play an important role in the final image of a specific pulse sequence. For example, FFE is faster than TSE with lower resolution and lower SNR. Increasing the scanning parameters, such as the number of signal averages (NSA), or the turbo factor (number of echoes received during one repetition time), increases the SNR. This enhancement of image quality requires a longer scanning time.

Further analysis is recommended to enhance the compatibility of the motor. Motor-induced image artifacts should be classified, and motor-induced geometric distortions in images should be quantified. In addition, other interactions of the USM and scanner such as the magnetic field’s applied force to the motor and the temperature increase of the motor should be characterized. Smart USMs can be developed by integrating their strong capabilities with enhanced performance to prevent compatibility issues.

## Conclusions

The presence of the motor results in SNR degradation of the MR images when the motor is on or off. The SNR loss is approximately 10 dB when the motor is in the x and y orientations, but the SNR values are higher when the motor is oriented along x than in the other two orientations. The SNR value is a useful parameter to evaluate the impact of compensation factors such as the slice thickness or BW that are used for reducing image artifacts caused by the USM. There is a trade-off between SNR values and slice thickness reduction or bandwidth increment. In developing any MRI-compatible assisted device actuated by a USM, this study recommends the use of an approximately 3-mm slice thickness with minimum bandwidth to achieve optimized SNR values when a USM is operating close to (within approximately 40 mm) the region being imaged. Furthermore, The SNR can be enhanced by increasing the number of signal averages, but this is achieved only at the cost of increasing the scan duration.

In Table 3, results are reported only for a slice thickness of 3 mm for all four sequences (T1W, T2W, TSE, and FFE). The slice thickness of 3 mm is the most desirable, as larger thicknesses result in larger artifact sizes and smaller thicknesses result in a very low SNR. From this table, it is concluded that T1W is the optimal sequence when minimum artifact size is required, T2W when minimum acquisition duration is required, and TSE when minimum geometric distortion is required.

In conclusion, this research shows that the future design and application of smart USMs can be revolutionized through contributions made in this research as well as characterization of other interactions between the motor and the scanner.
